# Mixed-method approaches to strengthen economic evaluations in implementation research

**DOI:** 10.1186/s13012-018-0850-6

**Published:** 2019-01-11

**Authors:** Alex R. Dopp, Peter Mundey, Lana O. Beasley, Jane F. Silovsky, Daniel Eisenberg

**Affiliations:** 1 Department of Psychological Science, University of Arkansas, 216 Memorial Hall, Fayetteville, AR 72701 USA; 2 Department of Pediatrics, University of Oklahoma Health Sciences Center, 940 NE 13th Street, Nicholson Tower, Suite 4900, Oklahoma City, OK 73104 USA; 3 Department of Human Development and Family Science, Oklahoma State University, 233 Human Sciences, Stillwater, OK 74078 USA; 4 Department of Health Management and Policy, University of Michigan School of Public Health, 1415 Washington Heights, Ann Arbor, MI 48109 USA

**Keywords:** Economic evaluation, Mixed methods, Health economics, Cost-effectiveness, Cost-benefit

## Abstract

**Background:**

Guidance from economic evaluations on which implementation strategies represent the best return on investment will be critical to advancing the Triple Aim of health care: improving patient care and population health while minimizing per-capita cost. The results of traditional (quantitative) economic evaluations are limited by a remaining “qualitative residual” of contextual information and stakeholders perspectives, which cannot be captured by monetary values alone and is particularly prevalent in implementation science research. The emergence of qualitative methods for economic evaluation offers a promising solution.

**Main body:**

To maximize the contributions of economic evaluations to implementation science, we recommend that researchers embrace a mixed-methods research agenda that merges traditional quantitative approaches with innovative, contextually grounded qualitative methods. Such studies are exceedingly rare at present. To assist implementation scientists in making use of mixed methods in this research context, we present an adapted taxonomy of mixed-method studies relevant to economic evaluation. We then illustrate the application of mixed methods in a recently completed cost-effectiveness evaluation, making use of an adapted version of reporting standards for economic evaluations.

**Conclusions:**

By incorporating qualitative methods, implementation researchers can enrich their economic evaluations with detailed, context-specific information that tells the full story of the costs and impacts of implementation. We end by providing suggestions for building a research agenda in mixed-method economic evaluation, along with more resources and training to support investigators who wish to answer our call to action.

## Background

In order to advance the three components of the Triple Aim in health care [1], there is a critical need for research that can identify the most impactful, efficient distribution of resources within health care organizations and systems. To date, implementation scientists have primarily focused on strategies to improve two components of the Triple Aim, (1) patient care (through uptake and adoption of evidence-based practices) and (2) population health (through scaling and sustainment of those practices), with scant attention to the third component: (3) minimizing per-capita cost [2, 3]. This results in a limited perspective because implementation efforts generally result in additional costs to agencies, which are often a critical barrier to implementation and sustainment of evidence-based practices [4, 5]. Guidance from economic evaluations on which implementation strategies represent the best return on investment will be critical to advancing the field. Herein, we argue that incorporation of mixed (i.e., qualitative and quantitative) methods in these evaluations will be necessary to maximize their contribution to implementation science.

### Economic evaluation of implementation

Traditional methods of economic evaluation in health care (see [6–8]) compare incremental differences in costs and outcomes—i.e., health-related efficacy or effectiveness data—among discrete clinical practices (e.g., two interventions in a clinical trial). Specific methods include cost analysis, which compares costs only; cost-effectiveness analysis, which compares costs to changes in a quantitative measure of health-related outcomes (or standardized outcomes, such as quality-adjusted or disability-adjusted life years, in the case of cost-utility analysis); benefit-cost analysis, which compares costs to monetized benefits of health-related outcomes (i.e., dollars to dollars); and budget impact analysis, which examines the consequences of an intervention on the budget of the agency that delivers it. These methods are all highly technical and quantitative, and have been applied most often to data from randomized clinical trials.

These traditional methods are certainly informative to implementation efforts (e.g., allow for consideration of an intervention’s economic effects when deciding whether to implement it). However, the methods become more complex and challenging when extended to implementation research. Such extensions involve comparing the costs of different implementation strategies to the outcomes (e.g., fidelity to or acceptability of the evidence-based practice; clinical symptoms) resulting from those strategies. As shown in the equation below, which compares the incremental costs and outcomes of two different implementation strategies, both the intervention and implementation strategy chosen must be considered when evaluating economic impact:

$$ \left({\mathrm{Cost}}_{\mathrm{Intervention}}+{\mathrm{Cost}}_{\mathrm{ImplementationStrategyA}}\right)-\left({\mathrm{Cost}}_{\mathrm{Intervention}}+{\mathrm{Cost}}_{\mathrm{ImplementationStrategyB}}\right) $$

vs.

$$ {\mathrm{Outcome}}_{\mathrm{InterventionW}/\mathrm{ImplementationStrategyA}}-{\mathrm{Outcome}}_{\mathrm{InterventionW}/\mathrm{ImplementationStrategyB}} $$

An additional challenge in economic evaluation is the “qualitative residual” [9] that often remains on the results of quantitative economic evaluations because of their limited ability to capture the contexts and stakeholders’ perspectives within which monetary values can be interpreted. This limitation is especially salient in implementation research because the outcomes [10] and costs [11, 12] are dependent on the context in which implementation takes place. Thus, in the equation above, each component carries its own qualitative residual. For example, the decisions made by clinic staff during implementation can influence intervention costs (e.g., by adding or dropping components), implementation costs (e.g., by allocating personnel and resources to implementation activities), and outcomes (e.g., poorly functioning clinics may need to expend more resources to achieve a desired level of implementation quality).

### Qualitative methods for economic evaluation

Recognizing limitations to exclusive use of quantitative economic evaluations, an increasing number of scholars outside of implementation science have advocated for the incorporation of qualitative data into those evaluations [13–15]. The traditions of qualitative research methods are as rich and varied as those of quantitative methods (see, e.g., [16, 17]). Techniques for data collection include individual interviews and focus groups designed to gather participant perspectives on a topic; site visits to observe where participants live, work, or play; review of records and other documents to glean insights about activities; or ethnographic field work in which the researcher is embedded within a community while collecting detailed observations. Analytic techniques apply a variety of perspectives to analyzing the words, themes, and language conventions that make up qualitative data, such as content analysis, thematic analysis, grounded theory, and in-depth case studies. The common thread through all qualitative methods is an emphasis on achieving a depth of understanding (often with a small sample of participants or groups) that captures the perspectives, experiences, or environments of certain individuals or groups.

Formal qualitative research occupies a small but growing place within the field of economics [18, 19]. Certainly, carefully done economic evaluations often contain informal qualitative components, even if those components are not identified as such. For example, the evaluators might develop data collection instruments based on careful discussions with practitioners or base their interpretations of the data on their familiarity with the organizations and settings involved. However, it is rare for these methods to be incorporated thoroughly into the analytical plan and process or to be formally documented. Moreover, few of the qualitative economic studies to date have focused on the economics of health care, let alone implementation specifically. Given that qualitative methods are ideally suited to provide the “thick description” [20] of contextual information needed for high-quality studies of implementation, rigorous application of qualitative methods provides rich information critical for economic evaluation that is unattainable by traditional, quantitative methods in isolation. Therefore, as described more next, a mixed-method approach is better suited for implementation research. More broadly, such an approach is also compatible with calls for an ethical imperative to include participants’ voices, using qualitative methods, in theoretical and empirical representations of the economic forces that shape their lives (including in health care) [19, 21].

#### A call for mixed-method economic evaluation

Despite their numerous strengths, we do not suggest that qualitative methods of economic evaluation should replace quantitative methods—which are well-established, rigorous, and have a long and impactful history of use. Instead, we recommend that implementation scientists begin to develop a research agenda around mixed-method economic evaluation. Mixed methods refer to a tradition that combines qualitative and quantitative data to address the same (or closely related) research questions [22, 23]. Combining the complementary strengths and perspectives of each research tradition allows for a better understanding of a research topic than either approach in isolation [23, 24] and provides an opportunity to derive emergent insights by merging multiple perspectives [25]. For these reasons, mixed methods are an essential component of “gold standard” studies in implementation science [10, 26].

Unfortunately, to date, virtually no research has combined quantitative and qualitative approaches in the economic evaluation of health services. We only located 165 results in a PubMed search on August 31, 2018, for the following terms: “((“economic eval*” OR “economic analysis” OR “cost-effect*” OR “cost-benefit” OR “cost-utility” OR “cost effect*” OR “cost benefit” OR “cost utility”) AND (“mixed method*” OR “mixed-method*”)) AND health.” A search with the same terms in EconLit returned only four results. Of the subset of these results that actually described an economic evaluation, most reported on a purely quantitative economic evaluation in the context of a larger mixed-method study (i.e., qualitative data were collected but were not used to answer questions about economic costs and impacts). For examples, see Heller et al.’s [27] evaluation of a management program for type I diabetes and Rise et al.’s [28] protocol for a randomized trial evaluating a modification to an occupational rehabilitation program. We only located one study, a benefit-cost analysis of the Australian acute care accreditation program [29], that explicitly integrated qualitative (focus groups, expert panels) and quantitative (cost information collected via surveys and semi-structured interviews, indicators of patient safety and quality of care extracted from administrative datasets) methods to identify, quantify, and validate the costs and benefits of accreditation.

Given a limited primary empirical literature from which to draw, our recommendations for mixed-method economic evaluations instead come from conceptual and methodological literature related to these topics, as well as our own experience conducting qualitative and mixed-method research. That experience includes an economic evaluation, described in more detail later, which is currently undergoing peer review.

#### Taxonomy of mixed-method economic evaluations

Palinkas and colleagues [10] previously developed a useful taxonomy that describes the arrangements of qualitative (“qual”) and quantitative (“quant”) methods within mixed-method implementation research studies. The major features of that taxonomy include the *structure* (e.g., sequential vs. simultaneous data collection and analysis; primary emphasis on qual methods, quant methods, or both equally), *function* (i.e., what is achieved by combining qual and quant data), and *process* (i.e., how they are combined) of mixed methods within the study. Table 1 presents an adapted version of that taxonomy that is specific to mixed-method economic evaluations. Our intent in creating this taxonomy was to aid implementation researchers in (a) conceptualizing an agenda of mixed-methods economic evaluation that spans the full breadth of potential mixed methods, and (b) selecting the appropriate study design when planning a given mixed-method economic evaluation.

**Table 1:** Taxonomy of mixed method designs for economic evaluation

Element	Category^a^	Definition^b^
Structure		
Sequential	Qual ➔ QUAN	Sequential collection and analysis of quantitative and qualitative data, beginning with qualitative data, for primary purpose of testing the economic impact of an implementation activity, e.g., *collect qualitative data about implementation costs to inform the design*, *execution*, *or analysis of a subsequent economic evaluation*.
	QUAN ➔ Qual	Sequential collection and analysis of quantitative and qualitative data, beginning with quantitative data, for primary purpose of testing the economic impact of an implementation activity, e.g., *after an economic evaluation of an implementation initiative*, *collect qualitative data about the value generated by implementing the target evidence*-*based practice*.
Simultaneous	Qual + QUAN	Simultaneous collection and analysis of quantitative and qualitative data for primary purpose of testing the economic impact of an implementation activity, e.g., *use both qualitative and quantitative data to perform cost*-*benefit analysis calculations for an implementation initiative*.
	QUAN + QUAL	Simultaneous collection and analysis of quantitative and qualitative data, giving equal weight to both testing the economic impact of an implementation activity and exploration/hypothesis generation about its economic costs and impacts, e.g., *while measuring costs as an implementation outcome*, *collect exploratory qualitative data about factors that increase or decrease implementation costs in different settings*.
Function		
	Convergence	Using both types of methods to answer the same question, through: (a) Comparison of results to see if they reach the same conclusion [MERGE two datasets together], e.g., *triangulation to validate quantitative measures of implementation costs using qualitative data*. (b) Converting a dataset from one type into another [CONNECT—transform] by quantifying qualitative data (e.g., *counting the number of times different categories of impact are mentioned*) or qualifying quantitative data (e.g., *extracting types of costs from a budget spreadsheet*).
	Complementarity	Using each type of method to answer a related question or series of questions for purposes of: Evaluation [EMBED one study within another so that one type of data provides a supportive role to the other dataset], e.g., *using quantitative data to evaluate costs and impacts and qualitative data to evaluate the processes through which those costs and impacts arose* (*was the implementation process efficient*?). Elaboration [CONNECT—elaborate, have one dataset build upon another dataset], e.g., *when comparing the cost*-*effectiveness of implementation strategies*, *use qualitative data to provide depth of understanding and quantitative data to provide breadth of understanding*.
	Expansion	Using one type of method to answer questions raised by the other type of method [CONNECT—expand], e.g., *collecting follow*-*up qualitative data to explore why an implementation strategy was not cost*-*beneficial in a certain setting*.
	Development	Using one type of method to answer questions that will enable use of the other type of method to answer other questions [CONNECT—initiate], e.g., *develop plan for measuring costs or conducting sensitivity analyses based on qualitative data*.
	Sampling	Using one type of method to define or identify the participant sample for collection and analysis of data using the other type of method [CONNECT—sample], e.g., *selecting interview informants to provide perspectives on costs and impacts based on responses to a survey questionnaire*.

Adapted from Palinkas et al. [10]

^a^*QUAL or Qual* qualitative, *QUANT or Quant* quantitative, upper- or lowercase indicates whether the method was primary/dominant versus secondary/subservient, respectively

^b^Rather than presenting the process elements separately in the table, we note each one that is relevant in brackets (with the type of process in uppercase letters [AS SUCH]) within the definition for a given function element (i.e., indicating the processes by which that function is achieved)

Our adapted taxonomy differs from the original in three key ways. First, because economic evaluation is ultimately focused on quant questions (e.g., amount of $) and hypothesis testing, a mixed-method economic evaluation is best described as a “pure” (i.e., equal emphasis on qual and quant) or “quant-dominant mixed” study [23]. Therefore, we excluded structural categories described by Palinkas et al. [10] in which qual methods were dominant. Second, we modified the definitions from the original taxonomy to include language and examples specific to economic evaluation. Note that in this taxonomy, qual data refers to information about the types of costs and impacts for an implementation activity, the contextual factors that influenced those costs and impacts, and their relative importance or priority. In contrast, quant data include any numeric information on implementation-related costs and impacts, such as monetary amounts, utilization frequency counts, or scores on a quantitative measure of symptoms. Finally, for simplicity of presentation, we combined the function and process dimensions of the taxonomy because they are closely aligned (i.e., the original taxonomy [10] described which functions are achieved through each process in addition to separately defining the functions).

### Illustrative example

Recently, a subset of the present authors completed a cost-effectiveness evaluation of the implementation of Problematic Sexual Behavior–Cognitive-Behavioral Therapy (PSB-CBT) at six provider agencies nationwide (Dopp A, Mundey P, Silovsky J, Hunter M, Slemaker A.: Economic value of community-based services for problematic sexual behaviors in youth: a mixed-method cost-effectiveness analysis, under review). PSB-CBT is a community-based, group-format treatment that has demonstrated significant effects on problematic sexual behavior in youth ages 7 to 14 (see [30]). This study provides a unique opportunity to illustrate, in detail, the mechanics of a mixed-method economic evaluation in an implementation study. Table 2 describes the study using the Consolidated Health Economic Evaluation Reporting Standards [31], and includes information about how mixed methods informed each item of the evaluation. We omitted some items because they were not relevant to our study or were non-methodological, and we only briefly mentioned items that did not incorporate qual data in our study. For example, our six participating agencies (“Setting and Location”) were selected based on funder decisions (whereas a mixed-method study with a sampling function might use qualitative data to select agencies). Moreover, it is beyond the scope of this article to provide guidance on all technical aspects of economic evaluation (e.g., perspective, discount rate), and many excellent resources already exist for quantitative economic evaluations in health care [6–8].

**Table 2:** Illustrative example: mixed-method cost-effectiveness evaluation of problematic sexual behavior-cognitive behavioral therapy

Section/item	No.^a^	CHEERS guideline	How met in illustrative example study^b^
Title and abstract			
Title	1	Identify the study as an economic evaluation.	Title: *Economic value of community*-*based services for problematic sexual behaviors in youth: A mixed*-*method cost*-*effectiveness analysis*.
Abstract	2	Provide a structured summary of the study.	We note that design, interpretation of quantitative analyses were informed by qualitative themes; both themes, quantitative threshold indicated that results represented cost-effective values.
Introduction			
Background and objectives	3	Provide an explicit statement of the broader context for the study. Present the study question and its relevance for health policy or practice.	Ability of treatments to reduce social, economic impacts of problematic sexual behavior is poorly understood. We used mixed-method cost-effectiveness analysis to compare costs of implementing PSB-CBT with clinical outcomes.
Method			
Target population and subgroups	4	Describe characteristics of the base case population and subgroups analyzed.	413 youth who received PSB-CBT at 1 of 6 provider agencies that implemented a PSB-CBT program between 2011 and 2015. We compared agencies that did vs. did not provide intensive individual services, did vs. did not incur indirect costs of implementation.
Setting and location	5	State relevant aspects of the system(s) in which the decision(s) need(s) to be made.	Agencies received grants from U.S. OJJDP to implement PSB-CBT, achieved adequate fidelity.
Study perspective	6	Describe the perspective of the study.	Perspective of agencies implementing PSB-CBT.
Comparators	7	Describe the interventions or strategies being compared and state why they were chosen.	PSB-CBT: cognitive-behavioral treatment model, with concurrent groups for youth and caregivers.
Time horizon	8	State and justify the time horizon(s) over which costs and consequences are evaluated.	Costs were measured for 6-month period. Outcomes were measured from 2011 to 2015.
Discount rate	9	Report and justify the choice of discount rate(s) used for costs and outcomes.	N/A—all costs were measured within a period of less than one year (i.e., 6 months).
Choice of health outcomes	10	Describe what outcomes were used as the measure(s) of benefit in the evaluation and their relevance for the study.	Caregiver-report measures of problematic sexual behaviors, nonsexual emotional and behavior problems (also self-reported), and traumatic stress symptoms.
Measurement of effectiveness	11a	Describe fully the design features of the effectiveness study.	Estimated pre-post changes in health outcome measures, expressed using effect size Cohen’s *d*.
Estimating resources and costs	13a	Describe approaches and data sources used to estimate and value resource use associated with the alternative interventions.	Collected quantitative cost surveys from participating agencies, covering costs related to running a PSB-CBT program. Cost survey was designed based on themes from qualitative interviews.
Currency, price date, and conversion	14	Describe methods for adjusting estimated unit costs to a common currency and price date.	Converted all monetary values to 2017 U.S. dollars (national average).
Analytic methods	17	Describe all analytical methods supporting the evaluation, including methods for handling population heterogeneity and uncertainty.	Calculated CERs as the per-youth cost of PSB-CBT divided by observed effect size for each health outcome. Compared CERs to a cost-effectiveness threshold of $8333. Used expertise plus qualitative themes to identify plausible range of values for key sources of uncertainty, variability; for sensitivity analyses, calculated CERs across those ranges of values.
Results			
Study parameters	18	Report the values, ranges, references, and, if used, probability distributions for all parameters. Report reasons or sources for distributions used to represent uncertainty where appropriate.	Median cost of $3527. Large to moderate (*d*s = 0.72–1.99) improvements on health outcome measures. We conducted four sensitivity analyses (described under 20a and 21).
Incremental costs and outcomes	19	For each intervention, report mean values for estimated costs and outcomes, as well as mean differences between the groups. If applicable, report incremental cost-effectiveness ratios.	CERs ranged from $1772 to $4899, indicating cost-effectiveness. Qualitative themes also indicated that PSB-CBT has valuable impact on families, society that is worth the cost.
Characterizing uncertainty	20a	Describe the effects of sampling uncertainty and methodological assumptions for the estimated incremental cost and effectiveness parameters.	Calculated CERs across plausible range for improvements on health outcome measures (95% CIs) and training costs (including vs. excluding initial training). All cost-effective except minimum improvement for traumatic stress.
Characterizing heterogeneity	21	If applicable, report differences in costs, outcomes, or cost effectiveness explained by variations between subgroups.	Calculated CERs for agencies that did vs. did not provide intensive individual services, indirect costs. All cost-effective except for agency with regular supplemental individual services.
Discussion			
Discussion	22	Summarize key study findings. Discuss limitations and generalizability of the findings; how the findings fit with current knowledge.	We note that the results of our qualitative interviews informed and supported the validity of our quantitative analyses.

*CER* cost-effectiveness ratio, *CHEERS* Consolidated Health Economic Evaluation Reporting Standards [30], *PSB*-*CBT* Problematic Sexual Behavior–Cognitive-Behavioral Therapy, *OJJDP* Office of Juvenile Justice and Delinquency Prevention. All monetary values are reported in 2017 U.S. dollars

^a^We omitted items 11b (synthesis-based measurement of effectiveness), 12 (preference-based outcomes), 13b (model-based evaluation), 15 and 16 (decision-analytic model and its assumptions), and 20b (characterizing uncertainty for model-based evaluation) because they were not applicable to the illustrative example study, and items 23 (source of funding) and 24 (conflicts of interest) because they are not methodological

^b^The illustrative example study (Dopp A, Mundey P, Silovsky J, Hunter M, Slemaker A.: Economic value of community-based services for problematic sexual behaviors in youth: a mixed-method cost-effectiveness analysis, under review)

Our evaluation benefitted from the use of mixed methods in two key ways. First, we took a Qual ➔ QUAN approach (*development* function, *connect*-*initiate* process; see Table 1) to create a survey of costs incurred during implementation of PSB-CBT. We developed the survey items based on qual data from interviews with 59 therapists, administrators, and external stakeholders from the agencies implementing PSB-CBT, ensuring broad coverage of costs that included staff activities, training expenses, number of youth served, and proportion of activities billed to various sources. Similarly, we planned several sensitivity analyses—which examine the influence of variation in model parameters on the findings of an economic evaluation [32]—using qual data about agency-specific contextual factors that affected implementation. For example, interviewees at an agency that regularly provided intensive individual services to youth in the PSB-CBT program noted higher costs, so we examined the impact of providing such services on cost-effectiveness and found that PSB-CBT was no longer cost-effective under those conditions. Of course, use of qualitative data in our evaluation design also introduced new challenges. In particular, interviewees described in detail how avoided expenses from alternatives to PSB-CBT (e.g., residential treatment, juvenile detention) were a key benefit of the program. However, when we asked program administrators during the cost survey to quantify savings from such avoided expenses, we found that administrators were unable to provide specific monetary values from the relevant agencies who would incur said costs. Potential cost-savings at the community rather than individual agency level complicates economic evaluation, with findings greatly restricted when relying on quan data alone.

Second, we used a QUAN + Qual approach to analyze and interpret our findings regarding the cost-effectiveness of PSB-CBT. Specifically, we used qual themes to validate conclusions from our quantitative cost-effectiveness ratios, representing a *convergence* function and *merge* process (see Table 1). This proved critical because, in the absence of quantitative data on the value of PSB-CBT outcomes (vs. alternative placements), we had to derive a quantitative threshold for cost-effectiveness from existing literature (detailed in, Dopp A, Mundey P, Silovsky J, Hunter M, Slemaker A.: Economic value of community-based services for problematic sexual behaviors in youth: a mixed-method cost-effectiveness analysis, under review). That threshold suggested that costs of up to $8333 per one-unit improvement in youth symptoms were cost-effective, but we needed a way to validate the threshold. We therefore examined themes from the qualitative interviews, in which respondents indicated that PSB-CBT added considerable value to families and society by providing a vital service that kept youth with PSB in the community, enhanced public safety, and was less expensive than traditional services for this population. These findings were consistent with the quantitative results, in which PSB-CBT was cost-effective under almost all conditions, thus allowing us to triangulate the conclusion that PSB-CBT has a valuable impact that is worth the cost of the program.

## Conclusions

In this article, we have recommended that implementation scientists embrace a mixed-method research agenda for economic evaluation, provided a taxonomy of mixed-method studies relevant to economic evaluation, and illustrated the application (and reporting) of these methods by presenting a recently completed study. Through incorporation of qualitative methods, implementation researchers can strengthen their economic evaluations with rich, contextually grounded stories that facilitate the interpretation (and actionability) of their results.

Of course, many challenges and unanswered questions remain in this area of research. We hope that other implementation researchers will use the proposed taxonomy and reporting standards to generate a more robust empirical research base. We also encourage those researchers to build on and modify the taxonomy and reporting standards; our example study had some notable limitations (e.g., lack of a comparison group) that may have led to concomitant limitations in the tools that we have developed thus far. Rigorous engagement with the proposed research agenda by many experts—working across a variety of implementation strategies, settings, and target evidence-based practices—will be necessary to reach scientific consensus on best practices in mixed-method economic evaluation. Across these various research efforts, examples of questions that could advance implementation science (while providing opportunities to explore and further refine mixed methods for economic evaluation) include:

1. What are the full economic costs and consequences of alternative implementation strategies and health services? These types of questions will extend traditional lines of economic evaluation research into the implementation science space. As in evaluation of other implementation outcomes, it will be critical to position findings within the contextual information and stakeholder perspectives provided by qualitative methods. Such information could be particularly valuable for understanding the economics of long-term sustainment of evidence-based practices following initial implementation, given the complex and dynamically changing factors involved.
2. How do the economic costs and consequences of implementation vary as a function of systemic and contextual factors (e.g., size of the organization, implementation climate)? This could be an excellent opportunity for simulation modeling and systems science approaches to economic evaluation [33], in which qualitative data could richly inform specification of the quantitative models.
3. What are the major sources of uncertainty when estimating the economic impact of implementation efforts, and how should those sources be accounted for? For instance, in implementation studies, it may be unclear to what extent start-up costs (e.g., training) will recur in the future (e.g., to provide refreshers to current personnel or to train new providers when turnover occurs). Another source of complexity is what perspective the economic evaluation should take (i.e., to whom are the costs and impacts incurred?), given the numerous stakeholder perspectives often represented in implementation efforts (e.g., when a mental health organization pays to implement an intervention that produces benefits in another sector, such as child welfare or criminal justice). The various scenarios or values to be represented in a sensitivity analysis [32] can be difficult to determine without a firm understanding of the qualitative context.
4. What is a reasonable return on investment for implementation and service outcomes; e.g., How much should we spend to increase fidelity to PSB-CBT (implementation outcome) or decrease average wait time to receive care (service outcome)? Such thresholds are not currently available in health economics literature (which has focused on returns on investment for clinical outcomes), presenting challenges for implementation researchers (see e.g., [34]). Incorporation of qualitative data into the development of such thresholds would likely strengthen their usefulness and credibility.
5. What are the best ways to address ethical issues introduced by using mixed methods in economic evaluations of implementation efforts? Inclusion of participant perspectives via qualitative methods certainly advances principles of justice and respect for persons [19, 21], but the level of detail captured by qualitative data also results in increased risks to participants [35]. For instance, collection of detailed qualitative information about implementation costs could threaten confidentiality by increasing the likelihood that participants are individually identifiable, as well as increase the potential harms of a breach in confidentiality (e.g., proprietary information could result in financial or legal ramifications in the event of a breach). It will be important to consider what types of training and guidelines will be necessary for researchers with a background in economic evaluation to learn and use established ethical practices for qualitative research (see [16, 17]). We anticipate that relevant topics might include confidentiality (e.g., de-identifying narrative data using pseudonyms and generic language) and data integrity (e.g., ensuring complete [non-selective] reporting of data, reporting quotations in context, determining when enough data have been collected to draw robust conclusions), among others.

We close by acknowledging that our proposed agenda will require researchers to continue pushing the boundaries of the interdisciplinary team science approaches that are already common—yet remain challenging—in health services research [10, 26, 36]. Mixed-method economic evaluations will require health services researchers to develop understanding of economic evaluation [2, 3] and for economists to develop understanding of qualitative and mixed methods [18, 19]. Thus, we hope to see an increase in resources (e.g., toolkits, formal coursework, mentored research programs) that support the development of researchers who combine qualitative and quantitative perspectives in economic evaluations. Such training would add to the growing plethora of implementation science resource initiatives [37]—paving the way for more innovative, contextually valid, and impactful studies to advance all aspects of the Triple Aim in health care [1].

## Abbreviations

CER: Cost-effectiveness ratio
CHEERS: Consolidated Health Economic Evaluation Reporting Standards
OJJDP: Office of Juvenile Justice and Delinquency Prevention
PSB-CBT: Problematic Sexual Behavior–Cognitive-Behavioral Therapy
QUAL or Qual: Qualitative
QUANT or Quant: Quantitative
